# An international multi-site study to evaluate the analytical and clinical performance of the quantitative high-throughput Alinity m CMV assay

**DOI:** 10.1128/spectrum.02009-24

**Published:** 2025-08-19

**Authors:** Julie W. Hirschhorn, Mark M. Sasaki, Yan Chin Tai, April Kegl, Tanjina Akter, Tanisha Dickerson, Momka Narlieva, Nhi Nhan, Tianxi Liu, Eriel Thornton, Patricia Jim, Ya-Jhu Lin, Li-An Wu, Tzu-Hsuan Hsieh, Po-Wei Huang, Jen-Fu Hsu, Stephen Young, Danijela Lucic, D. Yitzchak Goldstein, Chung-Guei Huang

**Affiliations:** 1 Department of Pathology and Laboratory Medicine, Medical University of South Carolina, Charleston, South Carolina, USA; 2 Molecular Diagnostics at Abbott, Des Plaines, Illinois, USA; 3 Abbott Laboratories (Singapore) Pte. Ltd., Singapore, Singapore; 4 Department of Pathology, Montefiore Medical Center, Bronx, New York, USA; 5 TriCore Reference Laboratories 159777 , Albuquerque, New Mexico, USA; 6 Department of Laboratory Medicine, Linkou Main Branch, Chang Gung Memorial Hospital, Taoyuan, Taiwan; 7 Department of Nursing, Chang Gung University of Science and Technology 63113, Taoyuan, Taiwan; 8 Research Center for Emerging Viral Infections, Department of Medical Biotechnology and Laboratory Science, Chang Gung University, Taoyuan, Taiwan; 9 Department of Neonatology, Linkou Chang Gung Memorial Hospital 38014, Taoyuan, Taiwan; 10 School of Medicine, College of Medicine, Chang Gung University, Taoyuan, Taiwan; 11 School of Medicine, National Tsing Hua University 34881, Hsinchu, Taiwan; Oklahoma State University College of Veterinary Medicine, Stillwater, Oklahoma, USA

**Keywords:** nucleic acid amplification test, transplant, CMV, viral load monitoring, high-throughput diagnostic

## Abstract

Cytomegalovirus (CMV) has a high prevalence in the general population and lifelong latency once an individual is infected. Primary infection or reactivation of CMV in immunocompromised individuals can lead to serious illness, organ failure, and death. Precise and accurate quantification of CMV is a valuable tool to support clinical decision-making and prevent adverse outcomes in transplant recipients. This international multicenter study evaluated the analytical performance of the Alinity m CMV assay using a commercially available panel (2.30 to 6.60 Log IU/mL) and clinical performance compared to the Abbott RealTi*m*e CMV assay and Roche cobas CMV assay run on the cobas 6800 system using deidentified remnant plasma specimens. Analysis of analytical precision with the commercial panel revealed a %CV of ≤6.6% and a total SD of ≤0.18 Log IU/mL. Plasma specimens (*n* = 748) initially tested with the RealTi*m*e CMV assay and subsequently run on the Alinity m CMV assay demonstrated a correlation coefficient of 0.972 with a mean bias of −0.05 Log IU/mL. Comparison of the Alinity m CMV and cobas CMV assays utilizing a separate set of plasma specimens (*n* = 303) demonstrated a correlation coefficient of 0.979 with a mean bias of 0.09 Log IU/mL. The overall percent agreement between Alinity m CMV and RealTi*m*e CMV assay results and between Alinity m CMV and cobas CMV assay results at clinically relevant thresholds was ≥95.7% and ≥96.4%, respectively. The high precision, accuracy, and comparable clinical performance of the Alinity m CMV assay, combined with rapid results reporting, support its use in transplant patient management.

**IMPORTANCE:**

Cytomegalovirus (CMV) in transplant patients can lead to adverse health consequences. Viral load monitoring is important in post-transplant patient care. Clinical diagnostic laboratories utilize various molecular diagnostic platforms. This study evaluated the Alinity m CMV assay with two commercially available assays: the Abbott RealTime CMV and Roche cobas CMV assays. Our results demonstrated a strong correlation between Alinity m CMV and the comparator assays, ensuring comparable results across these platforms.

## INTRODUCTION

Human cytomegalovirus (CMV), also known as Herpesvirus 5, is a double-stranded DNA virus with high worldwide prevalence: 83% seroprevalence in the general population and 86% in transplant donors (1, 2). Transmission of CMV may occur through direct contact with infected fluid, saliva, vaginal fluid, semen, breast milk, and transplanted organs, or via blood transfusion (3). Primary CMV infection in immunocompetent individuals results in mild illness, including fever, sore throat, and fatigue (4–6); however, primary infection or reactivation of latent CMV can cause severe complications in newborns and immunocompromised individuals (1–3). As CMV is a ubiquitous virus with broad cellular tropisms that allow it to infect multiple organs, CMV infection is associated with significant morbidity and mortality in immunocompromised individuals and hematopoietic stem cell and solid organ transplant recipients (7–9).

The CMV serostatus of donor and recipient (D/R) is predictive of the risk of CMV infection after transplant (5). Seropositive recipients (R+) are at moderate risk of CMV infection after transplant, and R+ recipients receiving an organ from CMV seropositive donors (D+) have a higher risk than those receiving an organ from a negative donor (D−) (2, 7). Viral load quantitation plays a key role in aiding in the post-transplant diagnosis of CMV infection and in monitoring the response to treatment. Highly sensitive and specific quantitative nucleic acid amplification tests (QNATs) allow for accurate quantitation of viral DNA and are the preferred method for detection of CMV replication (5, 10–13). Despite the widely accepted clinical utility of CMV QNATs, there is currently no single viral load threshold for clinical intervention (11, 12). The challenge is that viral load can vary depending on the assay design, sample type, transplantation type, and degree of immunosuppression (12). Even with the World Health Organization (WHO) International Standard, which is intended to promote harmonization of test reporting, variation between assays is still seen. As such, the clinical threshold for CMV surveillance and monitoring after transplantation remains institution- and assay-specific (11, 12).

In the current study, we aimed to evaluate the analytical performance of the Alinity m CMV assay and to evaluate the clinical performance of the assay compared with two on-market CMV assays, the Abbott RealTi*m*e CMV assay and the Roche cobas CMV assay run on the cobas 6800 system, using four clinically relevant CMV thresholds.

## MATERIALS AND METHODS

### Molecular assays

The Alinity m CMV assay (Abbott Molecular Inc., Des Plaines, IL, USA) is a real-time polymerase chain reaction (PCR) quantitative test performed on the Alinity m system (Abbott Molecular Inc.). Alinity m is a high-throughput, fully automated analyzer with continuous and random-access capabilities. The Alinity m CMV assay uses dual primers and probes to amplify and detect two targets (UL34 and UL80.5) in the CMV genome. The Alinity m CMV assay has an analytical measuring range (AMR) of 1.48 Log IU/mL to 8.00 Log IU/mL for plasma specimens (14).

The RealTi*m*e CMV assay (Abbott Molecular Inc.) uses dual primers and probes to amplify and detect two targets (UL34 and UL80.5) in the CMV genome. This real-time PCR quantitative test is performed on the *m*2000 system (Abbott Molecular Inc.), which includes automated sample preparation on the *m*2000*sp* and PCR on the *m*2000*rt*. The RealTi*m*e CMV assay has an AMR between 1.70 Log IU/mL to 8.19 Log IU/mL (15).

The cobas CMV assay (Roche Molecular Systems, Inc., Branchburg, NJ, USA) is a quantitative nucleic acid test for use on the cobas 6800 system. The assay uses a primer and probe to amplify and detect a conserved region in the UL54 gene; the AMR of the assay is between 1.54 Log IU/mL and 7.00 Log IU/mL (16).

### Analytical performance assessment

Alinity m CMV assay sensitivity was assessed by testing 20 replicates at 1.48 Log IU/mL, prepared by diluting a CMV verification panel (Bio-Rad Laboratories, Hercules, CA, USA) in negative CMV plasma. Linearity was verified across a range of CMV concentrations from 2.30 Log IU/mL to 6.60 Log IU/mL using a commercially available CMV panel in plasma (Bio-Rad Laboratories). Assay precision was evaluated by testing 35 replicates of each panel member run over multiple days across three study sites: Chang Gung Memorial Hospital, Linkou (CGMH-LK, Taoyuan City, Taiwan), Montefiore Medical Center (MMC, Bronx, NY, USA), and Medical University of South Carolina (MUSC, Charleston, SC, USA).

Alinity m CMV reproducibility was assessed at the three sites (CGMH-LK, MMC, and MUSC) by evaluating the performance of the assay quality controls (QC, low-positive control [LPC], and high-positive control [HPC]). Forty-five replicates were tested each for the LPC and HPC.

### Clinical performance evaluation study design and specimens

A total of 748 remnant de-identified plasma specimens that were initially tested fresh on the RealTi*m*e CMV assay were either tested fresh (*n* = 404) or after storage at −70°C (*n* = 344) on the Alinity m CMV assay. RealTi*m*e CMV testing occurred at three testing sites in the US: MMC, MUSC, and the University of Washington Medicine Clinical Virology Laboratory (Seattle, WA, USA) as part of routine clinical care. Alinity m CMV testing was performed at MMC, MUSC, and Molecular Diagnostics at Abbott (MDx).

A separate set of 303 remnant de-identified specimens was initially tested fresh with the cobas CMV assay run on the cobas 6800 system as part of routine clinical care, stored at −70°C, and later tested with the Alinity m CMV assay. cobas CMV testing occurred at TriCore Reference Laboratories (Albuquerque, NM, USA) and CGMH-LK, and Alinity m CMV testing was performed at CGMH-LK and MDx.

All clinical specimens were anonymized before study initiation, and a coded identification number with no patient identifiers was assigned to each remnant specimen. At MMC, an honest broker maintained the key to the coded samples to be reviewed individually if any other clinical information became necessary. At MUSC, the study protocol was considered a quality improvement project and was not subject to institutional review as per MUSC operating procedure. The testing protocol at MDx and TriCore Reference Laboratories was approved by the WCG review board. The study protocol for testing conducted at MMC and CGMH-LK was approved by the institutional review boards of the respective institutions. The study was performed at all sites according to the principles of Good Clinical Practice and conducted in adherence with the Declaration of Helsinki.

Due to the lack of clinical guideline thresholds, institutions implement their own thresholds. Most commonly used thresholds across the institutions range from 250 IU/mL to 2,000 IU/mL which may be due to the studies which have demonstrated that viral load ≥250 IU/mL is associated with increased risk of mortality after hematopoietic cell transplantation; a viral load of 2,000 IU/mL (3.30 Log IU/mL) is the threshold used to initiate pre-emptive treatment; and a viral load >3.30 Log IU/mL is associated with end organ disease (17, 18). This study used the lowest viral threshold amongst the study sites, which was 250 IU/mL, and the change in the viral load of >0.5 Log10 IU/mL was used to represent clinically significant differences in DNAemia.

### Workflow evaluation

Testing with the Alinity m CMV assay was done retrospectively; therefore, the time from receipt of the samples to result reporting could not be assessed. Instead, the onboard turnaround time (TAT; from placement of the sample on the analyzer to result reporting) and processing TAT (from the sample aspiration to result reporting) on the Alinity m system were evaluated based on the automatic Alinity m documentation of timepoints for sample loading, sample aspiration, and result reporting.

### Statistical analysis

The following analysis was performed for each instrument and each panel member. The PROC MIXED procedure with the MIVQUE0 option in SAS was used to determine variance components for the model used in the analysis. The point estimates of the means, standard deviations (SD), and % coefficients of variance (CV) were reported. The SD and %CV were estimated for the within-day component, the between-day component, and the between-site component for each instrument and each panel member. All the effects were considered random for the analyses. Any negative variance components were set to zero for these calculations. Variance components were estimated based on the random effects analysis of variance model: Y = Mean + Site + Day + Error. The total assay variability was defined as the sum of the within-day (residual error) component, the between-day component, and the between-site component estimates of variability. The following statistics were reported: N, mean, within-day SD and %CV, between-day SD and %CV, between-site SD and %CV, Total SD, and %CV.

Agreement analysis was performed by categorizing the results into five viral load ranges as described in the Clinical performance section, based on Alinity m CMV and RealTi*m*e CMV or cobas CMV assay results. For each viral load threshold, the percent agreement and associated two-sided score 95% intervals (CIs) for clinical specimens were calculated. Relationships between quantitative values were studied by means of Deming regression. Bland-Altman analysis was performed to evaluate the differences in quantification between the assays.

All analyses were performed using PC SAS (version 9.4) software (SAS, Cary, NC, USA).

## RESULTS

### Analytical performance

The Alinity m CMV assay detected 100% (20/20) of a panel prepared at 1.48 Log IU/mL. Analytical linearity of the Alinity m CMV assay, using a commercially available verification panel ranging from 2.30 to 6.30 Log IU/mL, was 1.00 and inversely correlated with Ct (Fig. S1). Precision analysis of the Alinity m CMV assay demonstrated total %CV ≤6.6% and total SD of ≤0.18 Log IU/mL (Table 1). The mean bias of all panel members to the assigned target concentrations was 0.03 Log IU/mL. Quality control reproducibility of the assay, testing 45 replicates of HPC and LPC across three sites, had a total %CV of 1.5% and total SD of 0.09 Log IU/mL for HPC and total %CV of 3.6% and total SD of 0.12 Log IU/mL for LPC (Table 2).

**TABLE 1:** Precision of the Alinity m CMV assay (*n* = 45 replicates per panel)^*a*^

Target concentration (Log IU/mL)	Mean observed concentration (Log IU/mL)	Within-day component		Between-day component		Between-site component		Total^*b*^	
		SD	%CV	SD	%CV	SD	%CV	SD	%CV
2.30	2.36	0.11	4.8	0.00	0.0	0.09	4.0	0.15	6.2
2.60	2.71	0.18	6.5	0.00	0.0	0.03	1.0	0.18	6.6
3.60	3.61	0.05	1.3	0.02	0.6	0.01	0.4	0.06	1.5
4.60	4.60	0.09	1.9	0.09	1.9	0.00	0.0	0.12	2.6
5.60	5.61	0.05	0.9	0.02	0.4	0.00	0.0	0.05	1.0
6.60	6.59	0.05	0.7	0.08	1.2	0.00	0.0	0.09	1.4

^*a*^ CV, coefficient of variance; SD, standard deviation.

^*b*^ Total includes within-day, between-day, and between-site components.

**TABLE 2:** Reproducibility testing of Alinity m CMV quality controls across three laboratories (*n* = 45 replicates for each control)^*a*^

Sample ID	Target concentration (Log IU/mL)	Mean observed concentration (Log IU/mL)	Within-day component		Between-day component		Between-site component		Total^*b*^	
			SD	%CV	SD	%CV	SD	%CV	SD	%CV
HPC	5.92–6.09	5.89	0.09	1.5	0.00	0.1	0.02	0.3	0.09	1.5
LPC	3.22–3.35	3.27	0.00	0.0	0.12	3.6	0.00	0.0	0.12	3.6

^*a*^ CV, coefficient of variance; SD, standard deviation.

^*b*^ Total includes within-day, between-day, and between-site components.

### Clinical performance

**Fig 1:**
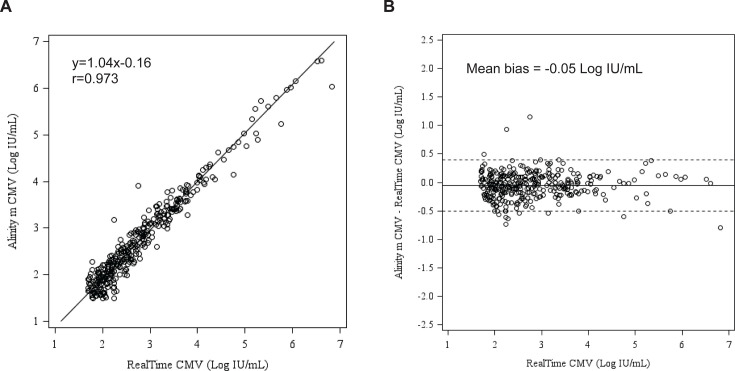
Clinical performance of the Alinity m CMV assay compared to the RealTi*m*e CMV assay with plasma specimens. Deming regression of (**A**) CMV levels showing correlation between the Alinity m CMV and the RealTi*m*e CMV assays. (**B**) Bland-Altman analysis showing mean bias between the Alinity m CMV and the RealTi*m*e CMV assays. Solid line indicates mean bias, dotted lines indicate ±1.96x SD.

A total of 748 plasma specimens were tested with the Alinity m CMV and RealTi*m*e CMV assays (Table 3). Overall percent agreement (OPA) at the four threshold concentrations of not detected (92.1%), <LLOQ (90.6%), 250 IU/mL (95.7%), and 2,000 IU/mL (97.7%) is shown in Table 4. Of the 168 results between the lower limit of quantitation (LLOQ) and <2.40 Log IU/mL by RealTi*m*e CMV, 10 were quantitated above the clinical threshold of 2.40 Log IU/mL, between 2.40 and 3.24 Log IU/mL. Of the 748 plasma samples tested by both RealTi*m*e CMV and Alinity m CMV, 342 were within the quantifiable range for both assays. The correlation coefficient between the two assays was 0.973 (Deming regression equation, y = 1.04x − 0.16), and the mean bias was −0.05 Log IU/mL (Bland-Altman analysis, Alinity m CMV—RealTi*m*e CMV, Fig. 1A and B).

**TABLE 3:** Agreement between Alinity m CMV and RealTi*m*e CMV assays (*n* = 748 plasma specimens)^*a*^

Alinity m CMV	RealTi*m*e CMV					
	Not detected	<LLOQ^*b*^	LLOQ to <2.40 Log IU/mL	2.40 to 3.30 Log IU/mL	>3.30 Log IU/mL	Total
Not detected	205	42	2	0	0	249
<LLOQ^*b*^	15	103	53	0	0	171
LLOQ to <2.40 Log IU/mL	0	15	103	22	0	140
2.40 to 3.30 Log IU/mL	0	0	10	84	8	102
>3.30 Log IU/mL	0	0	0	9	77	86
Total	220	160	168	115	85	748

^*a*^ LLOQ, lower limit of quantitation.

^*b*^ LLOQ used is the higher LLOQ of the two assays.

**TABLE 4:** Percent agreement and associated two-sided score 95% confidence interval (CI)^*a*^

Threshold	Percent agreement < threshold 95% score CI (n/N)	Percent agreement ≥ threshold 95% score CI (n/N)	Overall percent agreement 95% score CI (n/N)
Not detected	93.2 (89.1, 95.8) (205/220)	91.7 (89.0, 93.7) (484/528)	92.1 (90.0, 93.8) (689/748)
<LLOQ^*b*^	96.1 (93.6, 97.6) (365/380)	85.1 (81.1, 88.3) (313/368)	90.6 (88.3, 92.5) (678/748)
250	98.2 (96.7, 99.0) (538/548)	89.0 (83.9, 92.6) (178/200)	95.7 (94.0, 97.0) (716/748)
2,000	98.6 (97.4, 99.3) (654/663)	90.6 (82.5, 95.2) (77/85)	97.7 (96.4, 98.6) (731/748)

^*a*^ LLOQ, lower limit of quantitation.

^*b*^ The LLOQ used is the higher LLOQ between the two assays.

**Fig 2:**
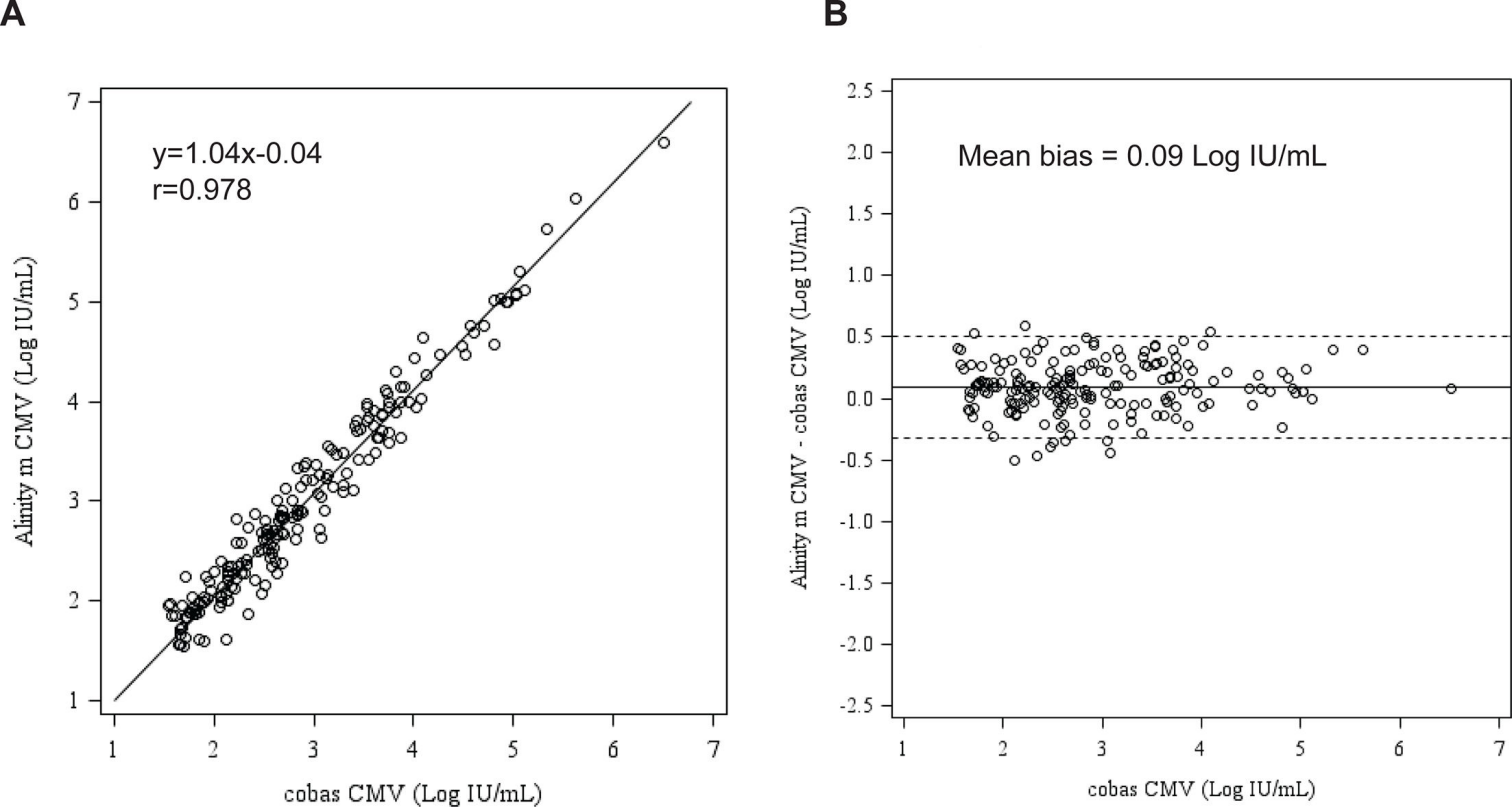
Clinical performance of the Alinity m CMV assay compared to the cobas CMV assay with plasma specimens. Deming regression of (**A**) CMV levels showing correlation between the Alinity m CMV and the cobas CMV assays. (**B**) Bland-Altman analysis showing mean bias between the Alinity m CMV and the cobas CMV assays. Solid line indicates mean bias, dotted lines indicate ±1.96x SD.

A total of 303 plasma specimens were tested with the Alinity m CMV and cobas CMV assays (Table 5). OPA at the four threshold concentrations of not detected (93.1%), <LLOQ (92.1%), 250 IU/mL (96.4%), and 2,000 IU/mL (97.0%) are shown in Table 6. Additionally, of the 70 results between LLOQ and 2.40 Log IU/mL by cobas CMV, five were quantitated above the clinical threshold of 2.40 Log IU/mL, between 2.40 and 3.24 Log IU/mL by Alinity m. Of the 303 plasma samples tested with both Alinity m CMV and cobas CMV, 186 were within the quantifiable range for both assays. The correlation coefficient between the two assays was 0.978 (Deming regression equation, y = 1.04x − 0.04), and the mean bias was 0.09 Log IU/mL (Bland-Altman analysis, Alinity m CMV—cobas CMV, Fig. 2A and B).

**TABLE 5:** Agreement between Alinity m CMV and cobas CMV assays (*n* = 303 plasma specimens)^*a*^

Alinity m CMV	cobas CMV					
	Not detected	<LLOQ^*b*^	LLOQ to <2.40 Log IU/mL	2.40 to 3.30 Log IU/mL	>3.30 Log IU/mL	Total
Not detected	42	16	0	0	0	58
<LLOQ^*b*^	5	30	7	0	0	42
LLOQ to <2.40 Log IU/mL	0	17	58	6	0	81
2.40 to 3.30 Log IU/mL	0	0	5	52	2^*d*^	59
>3.30 Log IU/mL	0	0	0	8^*c*^	55	63
Total	47	63	70	66	57	303

^*a*^ LLOQ, lower limit of quantitation.

^*b*^ LLOQ used is the higher LLOQ of the two assays.

^*c*^ Eight specimens quantitated between 2.40 to 3.30 Log IU/mL on cobas CMV were quantitated between 3.33 and 3.55 Log IU/mL on Alinity m CMV.

^*d*^ Two specimens quantitated between >3.30 Log IU/mL on cobas CMV were quantitated at 3.11 and 3.28 Log IU/mL on Alinity m CMV.

**TABLE 6:** Percent agreement and associated two-sided score 95% confidence interval (CI)^*a*^

Threshold	Percent agreement < threshold 95% score CI (n/N)	Percent agreement ≥ threshold 95% score CI (n/N)	Overall percent agreement 95% score CI (n/N)
Not detected	89.4 (77.4, 95.4) (42/47)	93.8 (90.1, 96.1) (240/256)	93.1 (89.6, 95.4) (282/303)
<LLOQ^*b*^	84.5 (76.6, 90.1) (93/110)	96.4 (92.7, 98.2) (186/193)	92.1 (88.5, 94.6) (279/303)
250	97.2 (93.7, 98.8) (175/180)	95.1 (89.8, 97.7) (117/123)	96.4 (93.6, 98.0) (292/303)
2,000	97.1 (94.2, 98.6) (238/245)	96.6 (88.3, 99.0) (56/58)	97.0 (94.5, 98.4) (294/303)

^*a*^ LLOQ, lower limit of quantitation.

^*b*^ The LLOQ used is the higher LLOQ of the two assays.

### Workflow analysis

The Alinity m system allows random continuous loading of Alinity m CMV assay samples, side-by-side with samples of other Alinity m assays that are processed simultaneously on the system. In this study, a total of 1,001 clinical specimens were tested on the Alinity m CMV at CGMH-LK, MDx, MMC, and MUSC. The median observed onboard TAT from sample placement to result reporting for Alinity m CMV was 2 hours 49 minutes (ranging from 2 hours 5 minutes to 5 hours 19 minutes). Median sample processing TAT from initiation of the test to result reporting was 1 hour 53 minutes (ranging from 1 hour 53 minutes to 1 hour 56 minutes).

The Abbott *m*2000 system is a batch analyzer capable of processing up to 96 tests in a standard 8 hour shift. Upon receipt of 100 samples into a laboratory, a single *m*2000 system can process 93% of the samples and three controls, with 7% of samples carried over to the next day (19).

Clinical specimens were processed for testing on the cobas CMV assay upon receipt or were batched prior to testing on the cobas 6800 system by the laboratory at CGMH-LK. Of the 253 specimens, 75.5% (191/253) were tested the same day they were received, 17.8% (45/253) specimens were tested the following day, and 6.7% (17/253) were tested 2 to 3 days after receipt. The median time from the receipt of the sample to result reporting was 7 hours and 54 minutes.

## DISCUSSION

In this multicenter evaluation, we found that the Alinity m CMV assay had a 100% detection rate at a CMV concentration of 30 IU/mL (1.48 Log IU/mL) and demonstrated high precision for quantifying CMV viral load across the dynamic range (2.30 to 6.60 Log IU/mL) with total SD ≤0.18 Log IU/mL. A single replicate at 2.60 Log IU/mL was quantitated at 3.44 Log IU/mL. Investigation of the cause was inconclusive, although sample handling error cannot be disregarded. Even with the inclusion of this outlier result, the mean bias of all panel members to the assigned target concentrations was 0.03 Log IU/mL. Our results are consistent with those of another multicenter study that reported a mean bias of 0.04 Log IU/mL and total SD of <0.28 Log IU/mL (15).

Clinical performance evaluation of the Alinity m CMV assay demonstrated a coefficient of correlation of 0.973 with the RealTi*m*e CMV assay and 0.978 with the cobas CMV assay, with mean bias of −0.05 Log IU/mL and 0.09 Log IU/mL, respectively. Discordant results between the Alinity m CMV and RealTi*m*e CMV assays (57/59, and the remaining two were <2.40 Log IU/mL) and the cobas CMV assay (21/21) were mostly seen for specimens with viral loads at the lower end of the quantitation range (not detected or <LLOQ) of the assays, and thus may not be clinically significant. The discordant results may be attributed to the imprecision of the assays at such a low viral load. The Third International Consensus Guidelines on the Management of Cytomegalovirus in Solid-organ Transplantation recommend that changes of at least 0.5 Log IU/mL be considered biologically significant changes in viral replication, and for viral loads <1,000 IU/mL, a greater variation (0.7 Log IU/mL) is permitted (11).

Though earlier reports showed high variability among CMV QNATs (20), our study showed satisfactory comparability between the Alinity m CMV assay and the RealTi*m*e CMV and cobas CMV assays. The high correlation between these assays may be attributed to the fact that all three assays are calibrated to the WHO International Standard for CMV QNAT (17, 18, 20). Some variations may be observed due to the difference in assay target selection, extraction chemistry, and calibration strategy between the Alinity m and cobas CMV assays; however, these differences were not evident in this study.

Specimen storage conditions did not appear to be a significant contributor to the discordant results, as Alinity m CMV testing was performed on both fresh and frozen specimens. Even for specimens stored for a median of 94 days (about 3 months) at −70°C prior to Alinity m CMV testing, no significant difference was observed between the results from the Alinity m CMV and cobas CMV assays.

There is no widely accepted clinical threshold for CMV viral load in the diagnosis and surveillance of CMV infection in the post-transplant setting (11, 12). We compared the performance of the three CMV QNATs by using four previously investigated clinically relevant thresholds: a viral load ≥250 IU/mL (2.40 Log IU/mL) is associated with increased risk of mortality after hematopoietic cell transplantation; a viral load of 2,000 IU/mL (3.30 Log IU/mL) is the threshold used to initiate pre-emptive treatment; and a viral load >3.30 Log IU/mL is associated with end organ disease (17, 18). The OPA at these thresholds was ≥95.7% between the Alinity m CMV and Abbott RealTime CMV assays and was ≥96.4% between the Alinity m CMV and cobas CMV assays. Due to the retrospective nature of this study, we were not able to evaluate the clinical impact of applying these thresholds.

The Alinity m system is a high-throughput analyzer whose continuous random-access capabilities are predicated on all Alinity m assays requiring the same TAT, regardless of the analyte being tested. This study was performed in a real-world setting where specimens for Alinity m CMV testing were run concurrently with other Alinity m assays. The median onboard TAT of 2 hours 49 minutes and median processing TAT of 1 hour 53 minutes observed with clinical specimens tested at MMC, MUSC, and CGMH-LK fell within the range observed by Obermeier et al., who evaluated TAT at eight multi-national sites, performing Alinity m HIV-1, HBV, HCV, STI, and HR HPV testing (21). The Alinity m provides faster result reporting than the Abbott RealT*im*e system, a batch analyzer with separate sample extraction on the *m*2000*sp* and amplification on the *m*2000*rt*, and the cobas 6800, another batch analyzer with a sample processing time of approximately 3 hours (19, 21).

An important strength of our study is that testing was performed across multiple sites, using multiple systems and different reagent lots, which controls for many of the possible operational and systemic variations that occur in the clinical laboratory. Limitations of our study include the use of surplus samples for assay comparison, resulting in insufficient volume to retest discordant results, and the inability to test samples concurrently with the two assays. In addition, there is no clinical threshold that applies to all institutions, thus limiting the comparison of the results.

Monitoring for CMV is critical for preventing adverse outcomes associated with viral reactivation, including graft rejection and secondary infections, and for improving graft and patient survival (11, 12). Precise CMV quantitation can help guide clinical decision-making and faster reporting of test results, with the fully automated Alinity m platform may have a significant impact on patient care. Taken together, this study supports the utility of the Alinity m CMV assay in transplant patient management.

## SUPPLEMENTAL MATERIAL

Fig. S1 spectrum.02009-24-s0001.eps Analytical performance of the Alinity m CMV assay.

Supplemental material spectrum.02009-24-s0002.docx Legend for Fig. S1.

ASM does not own the copyrights to Supplemental Material that may be linked to, or accessed through, an article. The authors have granted ASM a non-exclusive, world-wide license to publish the Supplemental Material files. Please contact the corresponding author directly for reuse.

## References

[1] Fowler K, Mucha J, Neumann M, Lewandowski W, Kaczanowska M, Grys M, Schmidt E, Natenshon A, Talarico C, Buck PO, Diaz-Decaro J. 2022. A systematic literature review of the global seroprevalence of cytomegalovirus: possible implications for treatment, screening, and vaccine development. BMC Public Health 22:1659. doi:10.1186/s12889-022-13971-7 36050659 PMC9435408

[2] Zuhair M, Smit GSA, Wallis G, Jabbar F, Smith C, Devleesschauwer B, Griffiths P. 2019. Estimation of the worldwide seroprevalence of cytomegalovirus: a systematic review and meta-analysis. Rev Med Virol 29:e2034. doi:10.1002/rmv.2034 30706584

[3] Centers for Disease Control and Prevention. 2020. About cytomegalovirus (CMV). CDC.

[4] Wreghitt TG, Teare EL, Sule O, Devi R, Rice P. 2003. Cytomegalovirus infection in immunocompetent patients. Clin Infect Dis 37:1603–1606. doi:10.1086/379711 14689339

[5] Grossi PA, Kamar N, Saliba F, Baldanti F, Aguado JM, Gottlieb J, Banas B, Potena L. 2022. Cytomegalovirus management in solid organ transplant recipients: a pre-COVID-19 survey from the working group of the European society for organ transplantation. Transpl Int 35:10332. doi:10.3389/ti.2022.10332 35812158 PMC9257585

[6] Razonable RR. 2020. Cytomegalovirus in solid organ transplant recipients: clinical updates, challenges and future directions. Curr Pharm Des 26:3497–3506. doi:10.2174/1381612826666200531152901 32473617

[7] Boeckh M, Geballe AP. 2011. Cytomegalovirus: pathogen, paradigm, and puzzle. J Clin Invest 121:1673–1680. doi:10.1172/JCI45449 21659716 PMC3083799

[8] Camargo J.F, Komanduri KV. 2017. Emerging concepts in cytomegalovirus infection following hematopoietic stem cell transplantation. Hematol Oncol Stem Cell Ther 10:233–238. doi:10.1016/j.hemonc.2017.05.001 28641094

[9] Griffiths P, Baraniak I, Reeves M. 2015. The pathogenesis of human cytomegalovirus. J Pathol 235:288–297. doi:10.1002/path.4437 25205255

[10] Camargo Jose F, Kimble E, Rosa R, Shimose LA, Bueno MX, Jeyakumar N, Morris MI, Abbo LM, Simkins J, Alencar MC, Benjamin C, Wieder E, Jimenez A, Beitinjaneh A, Goodman M, Byrnes JJ, Lekakis LJ, Pereira D, Komanduri KV. 2018. Impact of cytomegalovirus viral load on probability of spontaneous clearance and response to preemptive therapy in allogeneic stem cell transplantation recipients. Biol Blood Marrow Transplant 24:806–814. doi:10.1016/j.bbmt.2017.11.038 29217388

[11] Kotton CN, Kumar D, Caliendo AM, Huprikar S, Chou S, Danziger-Isakov L, Humar A, The Transplantation Society International CMV Consensus Group. 2018. The third international consensus guidelines on the management of cytomegalovirus in solid-organ transplantation. Transplantation 102:900–931. doi:10.1097/TP.0000000000002191 29596116

[12] Razonable R.R, Humar A. 2019. Cytomegalovirus in solid organ transplant recipients-guidelines of the American society of transplantation infectious diseases community of practice. Clin Transplant 33:e13512. doi:10.1111/ctr.13512 30817026

[13] Razonable RR, Inoue N, Pinninti SG, Boppana SB, Lazzarotto T, Gabrielli L, Simonazzi G, Pellett PE, Schmid DS. 2020. Clinical diagnostic testing for human cytomegalovirus infections. J Infect Dis 221:S74–S85. doi:10.1093/infdis/jiz601 32134488 PMC7057790

[14] Abbott Molecular. 2022. Alinity m CMV AMP kit [Package Insert]

[15] Abbott RealTime CMV. 2019. Abbott RealTime CMV [Package Insert]

[16] Roche. 2023. Cobas CMV quantitative nucleic acid test for use on cobas 5800/6800/8800 [Package Insert]

[17] Green ML, Leisenring W, Xie H, Mast TC, Cui Y, Sandmaier BM, Sorror ML, Goyal S, Özkök S, Yi J, Sahoo F, Kimball LE, Jerome KR, Marks MA, Boeckh M. 2016. Cytomegalovirus viral load and mortality after haemopoietic stem cell transplantation in the era of pre-emptive therapy: a retrospective cohort study. Lancet Haematol 3:e119–e127. doi:10.1016/S2352-3026(15)00289-6 26947200 PMC4914379

[18] Humar A, Gregson D, Caliendo AM, McGeer A, Malkan G, Krajden M, Corey P, Greig P, Walmsley S, Levy G, Mazzulli T. 1999. Clinical utility of quantitative cytomegalovirus viral load determination for predicting cytomegalovirus disease in liver transplant recipients. Transplantation 68:1305–1311. doi:10.1097/00007890-199911150-00015 10573068

[19] Lucic D, Jones S, Wiesneth R, Barry C, Webb E, Belova L, Dolan P, Ho S, Abravaya K, Cloherty G. 2013. Impact of the New Abbott mPLUS feature on clinical laboratory efficiencies of abbott RealTime assays for detection of HIV-1, Hepatitis C Virus, Hepatitis B Virus, Chlamydia trachomatis, and Neisseria gonorrhoeae. J Clin Microbiol 51:4050–4054. doi:10.1128/JCM.01672-13 24088850 PMC3838060

[20] Hayden RT, Preiksaitis J, Tong Y, Pang X, Sun Y, Tang L, Cook L, Pounds S, Fryer J, Caliendo AM. 2015. Commutability of the first world health organization international standard for human cytomegalovirus. J Clin Microbiol 53:3325–3333. doi:10.1128/JCM.01495-15 26269622 PMC4572535

[21] Obermeier M, Pacenti M, Ehret R, Onelia F, Gunson R, Goldstein E, Chevaliez S, Vilas A, Glass A, Maree L, Krügel M, Knechten H, Braun P, Naeth G, Azzato F, Lucic D, Marlowe N, Palm MJ, Pfeifer K, Reinhardt B, Dhein J, Joseph AM, Martínez-García L, Galán J-C. 2020. Improved molecular laboratory productivity by consolidation of testing on the new random-access analyzer Alinity m. J Lab Med 44:319–328. doi:10.1515/labmed-2020-0102

