# Supplementary material for: An international multi-site study to evaluate the analytical and clinical performance of the quantitative high-throughput Alinity m CMV assay
Source: Microbiol Spectr. 2025 Aug 19;13(10):e02009-24. doi: 10.1128/spectrum.02009-24 (PMC12502681; doi:10.1128/spectrum.02009-24)
Supplement: Supplemental material — Legend for Fig. S1. [file spectrum.02009-24-s0002.docx]

**Supplemental Figure Legends**

Supplemental Figure 1: Analytical performance of the Alinity m CMV assay. Linearity was established from 2.30 to 6.60 Log IU/mL using a commercially available verification panel. (A) Target concentration versus mean observed concentration. (B) Target concentration versus mean observed CT.
